# What gardens grow: Outcomes from home and community gardens supported by community-based food justice organizations

**DOI:** 10.5304/jafscd.2018.08A.002

**Published:** 2018-06-18

**Authors:** Christine M. Porter

**Affiliations:** University of Wyoming

**Keywords:** Home Gardens, Community Gardens, Public Health, Community Food Systems, Community Food Production, Food Justice, Community-based Organizations (CBOs), Community-based Participatory Research (CBPR), Food Dignity

## Abstract

Supporting home and community gardening is a core activity of many community-based organizations (CBOs) that are leading the food justice movement in the U.S. Using mixed methods across multiple action-research studies with five food justice CBOs, this paper documents myriad layers of benefits that gardening yields.

Our participatory methods included conducting extensive case studies with five CBOs over five years; quantifying food harvests with 33 gardeners in Laramie, Wyoming, and surveying them about other gardening outcomes (20 responded); and conducting feasibility studies for assessing health impacts of gardening with two of the five CBOs, both in Wyoming.

Analyses of these diverse data yielded four categories of gardening benefits: (1) improving health; (2) producing quality food in nutritionally meaningful quantities; (3) providing cultural services; and (4) fostering healing and transformation.

Examining these results together illustrates a breadth of health, food, and cultural ecosystem services, and social change yields of home and community food gardening in these communities. It also points to the need to support CBOs in enabling household food production and to future research questions about what CBO strategies most enhance access to and benefits of gardening, especially in communities most hurt by racism and/or insufficient access to fresh food.

## Introduction

Food gardening has become a mainstay of community-based food security, food justice and even obesity prevention initiatives in the United States (Gatto, Martinez, Spruijt-Metz, & Davis, 2017; Gonzalez, Potteiger, Bellows, Weissman, & Mees, 2016; Lawson, 2005; Saul & Curtis, 2013; Zanko, Hill, Estabrooks, Niewolny, & Zoellner, 2014). In addition, gardening has becoming increasingly popular overall in the U.S. For example, the Five Borough Farm project in New York City documented 700 community food gardens and farms in the city in 2011; then, in a second canvas three years later, they identified over 900 (Altman et al., 2014). Similarly, a National Gardening Association study found that household gardening increased by 17% over five years (2008–2013), including by 38% among lower-income households (≤S$35,000). Also, given the 63% increase in gardening among millennials, public interest in gardening seems unlikely to abate soon (National Gardening Association, 2014).

Given that a growing body of research suggests food gardening may offer a partial solution towards tackling a few of our most wicked social problems in the U.S.—including chronic disease, food insecurity, socioeconomic inequity, and shrinking social ties—this growth of food gardening in the U.S. is arguably a welcome trend and one potentially worthy of public support and investment. This paper briefly reviews the evidence base about the benefits of food gardening. It then shares results from research generated over five years via a wide range of mixed and participatory methods with five U.S. food-justice oriented, community-based organizations (CBOs), to answer the following research question: what was the range, quality and quantity of gardening outcomes in these communities, with support from these five CBOs?

As the first study in the U.S. to use multiple methods over multiple years with multiple communities and CBOs to document multiple forms of gardening yields, this research contributes an uniquely rich breadth and depth of data and analysis to the benefits-of-gardening literature.

## Literature Review

Growing literature about benefits of home and community gardening suggests that gardening improves health, produces meaningful amounts of food, and provides multiple forms of ecosystem services. A smaller body of work, observational or occasionally theoretical, also considers the role of gardening in social change.

### Health

Health benefits of gardening, that have been suggested by a mostly observational body of work, have included increasing fruit and vegetable intake (Alaimo, Packnett, Miles, & Kruger, 2008; Armstrong, 2000; Litt et al., 2011; Meinen, Friese, Wright, & Carrel, 2012; Twiss et al., 2003), fostering physical activity (Armstrong, 2000; Draper & Freedman, 2010; Park, Shoemaker, & Haub, 2009), reducing food insecurity (Baker, Motton, Seiler, Duggan, & Brownson, 2013; Corrigan, 2011; Stroink & Nelson, 2009), improving metal health (Austin, Johnston, & Morgan, 2006; Brown & Jameton, 2000; van den Berg, van Winsum-Westra, de Vries, & van Dillen, 2010; Wakefield, Yeudall, Taron, Reynolds, & Skinner, 2007), improving body mass index (Utter, Denny, & Dyson, 2016; Zick, Smith, Kowaleski-Jones, Uno, & Merrill, 2013), and increasing social capital (Alaimo, Reischl, & Allen, 2010; Armstrong, 2000; Twiss et al., 2003).

The quality of this evidence base is mixed. Few studies of health impacts of home and community gardening have control groups and we found none with randomized control groups. However, evidence should be available by 2022 from three randomized controlled trials in the U.S. that are currently recruiting. One is assessing health impacts of community gardens (University of University of Colorado at Boulder, Michigan State University, Colorado School of Public Health, University of South Carolina, Colorado State University, & Denver Urban Gardens, 2017). Two are assessing home food gardens (University of Wyoming et al., 2016; University of Alabama at Birmingham, Auburn University, & National Cancer Institute, 2016). (Note: the Growing Resilience trial emerged from the feasibility pilot studies reported in this paper.)

In the meantime, a recent meta-analysis of quantitative results from 22 garden studies confirms most of the health outcomes described above (Soga, Gaston, & Yamaura, 2017), with the notable exception of garden impacts on food insecurity, which was not examined in the included studies.

### Food

Though the literature above has provided only weak evidence about impacts of gardening on food security, a growing body of harvest quantification research suggests that gardeners harvest nutritionally and economically meaningful amounts of food (Algert, Baameur, & Renvall, 2014; Algert, Diekmann, Renvall, & Gray, 2016; CoDyre, Fraser, & Landman, 2015; Gittleman, Jordan, & Brelsford, 2012; Pourias, Duchemin, & Aubry, 2015; Smith & Harrington, 2014; Vitiello & Nairn, 2009; Vitiello, Nairn, Grisso, & Swistak, 2010). Also, a survey with 66 New York City gardeners found that gardens were the primary or secondary produce source for 90% of respondents who were food insecure (*n*=19), versus for 71% of the 47 gardeners who were food secure (Gregory, Leslie, & Drinkwater, 2016).

Overall, these findings indicate that it is plausible that successful food gardening would improve food security by provisioning nutritional meaningful quantities of food. Also, obviously, gardening yields a particular kind of food: fruits and vegetables. U.S. adults of all socioeconomic groups eat much less of these foods than recommended (Guenther, Dodd, Reedy, & Krebs-Smith, 2006), and to those struggling with low incomes (and some living in communities predominantly of color report price) availability and quality serve as barriers to consumption (Haynes-Maslow, Parsons, Wheeler, & Leone, 2013; Yeh et al., 2008). Successfully growing produce at home could help overcome some economic and geographic barriers to fresh vegetables or fruits.

### Other Ecosystem Services

Gardening provides ecosystem services, that is, benefits that people obtain from ecosystems, such as fiber, water filtering, and enjoyment (Millennium Ecosystem Assessment, 2005). These ecosystem services include “provisioning” benefits, in providing food and health outcomes described above.

Evidence suggests that gardening also yields “regulating” ecosystem services, via increasing climate and water quality, supporting soil formation, fostering nutrient cycling, and sustaining biodiversity (Altman et al., 2014; Calvet-Mir, Gómez-Baggethun, & Reyes-García, 2012; Cohen & Reynolds, 2015; Cohen, Reynolds, & Sanghvi, 2012).

The third set of ecosystem services that gardens provide are cultural (including spiritual), social, and recreational services. Growing food, including culturally relevant foods, has helped some communities maintain cultural connection and continuity (Companion, 2016; Hartwig & Mason, 2016). Several garden studies have found that participating in community gardens especially, has helped to build social capital and connectedness, self-efficacy, and civic engagement (Firth, Maye, & Pearson, 2011; Hartwig & Mason, 2016; Litt et al., 2011; Ober Allen, Alaimo, Elam, & Perry, 2008). For example, one case study with an urban gardening program found that it provided a, “social bridge to build community cohesion” (Gonzalez et al., 2016, p. 107). One scholar, examining the history of gardening in the U.S. and two community garden cases in San Fransico, suggests that such, “organized gardening projects,” serve to, “cultivate specific kinds of citizen-subjects” (Pudup, 2008). Pudup’s paper points to a fourth category of gardening outcomes, i.e., shaping society and promoting social change work (while also noting that such cultural services are not always inherently positive; see also Glover, 2004). The cultural ecosystem services that gardens yield might be seen as both foundations for and contributions to ways gardening also might foster social change.

### Social Change

Organizers and organizations in the U.S. food justice movement (Bradley & Herrera, 2016; Sbicca, 2012) extensively employ home and community gardening as part of anti-oppression and other transformational strategies for creating equity, health, sustainability, and/or food sovereignty (Broad, 2016; White, 2011a, 2011b, 2017; Winne, 2008, 2010). Others have also linked propagating seeds with promoting social change (Follmann & Viehoff, 2015; McKay, 2011; Nettle, 2014). This includes empowerment outcomes identified in case studies with Seattle community gardens (Hou, Johnson, & Lawson, 2009) and the Five Borough Farm action research project documenting benefits of community-based food production for, “making New York City a healthier and more socially connected, economically secure, and environmentally sustainable city” (Cohen, Reynolds, & Sanghvi, 2012, p. 9).

Of the four categories of gardening yields discussed here—health, food, cultural ecosystem services, and social change—social change outcomes are the widest reaching and also the most challenging to systematically identify, attribute, and assess. The aforementioned health, food, and cultural “services” from gardening plausibly enable and contribute to such larger social change. Indeed, in their analysis of case studies with four community garden groups in London, United Kingdom, two scholars find that these organized gardening projects foster, “prefigurative social change,” based on a shared practice of gardening rather than on strategic intention, “opening up new possibilities for being, seeing and doing” (Guerlain & Campbell, 2016, p. 220).

The research presented here adds to the garden outcome literature described above by examining results from a group of related studies using multiple research methods to identify and characterize yields from home and community gardening.

## Methods

This work originated with Food Dignity, a five-year action-research project about food system sustainability and security strategies employed by five food justice CBOs in the U.S. These CBO partners were Blue Mountain Associates (BMA) on Wind River Indian Reservation; Feeding Laramie Valley (FLV) in Laramie, Wyoming; Whole Community Project (WCP) in Ithaca, New York; East New York Farms! (ENYF) in Brooklyn, New York; and Dig Deep Farms (DDF) in the unincorporated areas of Ashland and Cherryland in the San Francisco Bay area of California.

Results in this paper derive from Food Dignity and other collaborative action-research projects conducted with these five CBOs between 2011 and 2016. As described in more detail below, we used a wide array of methods in three relatively distinct but related research endeavors:

1. Developing deep case studies, or rigorous stories, with and about the work of each of the five CBOs partners in Food Dignity.
2. Quantifying garden yields via gardener-researchers weighing every harvest and assessing other forms of outcomes via surveys with the gardeners. This was a sub-project of Food Dignity conducted in partnership with FLV. We called it “Team GROW.”
3. Implementing controlled trial feasibility pilot studies to assess the health impacts of gardens with FLV and BMA. We called these pilots “Growing Resilience.”

I was the project director and principal investigator for all of these studies.

### Food Dignity Case Study Methods

The main research method in Food Dignity is rigorous storytelling, or deep case studies, to document the context, history, and practices of the five CBO collaborators. Our methods included conventional case study approaches (Yin, 2009). We conducted 150 stakeholder interviews, over five years of insider and outsider participation and observation, and extensive primary and secondary document analysis. We created collaborative pathway models with each CBO, which illustrate the theories of change underlying a CBO’s activities by linking them to expected outcomes (Hargraves & Denning, 2017). We also produced first-person digital stories about our journeys to food justice and Food Dignity work, 12 of which were created by CBO partners (Food Dignity, 2015).

For this research, I sifted back through this enormous data set to identify outcomes from gardening. I coded the five CBO collaborative pathway models and the transcripts of the 12 community-authored digital stories for gardening-related themes, extracting every mention of the word “garden” and its variations for further analysis. I focused particularly on these because they are products in their own right, which CBO partners in Food Dignity have used to codify their work. I also electronically searched for variations on the word “garden” to identify all potentially relevant passages from our collection of interview transcripts and our field notes from participation and observation. I then analyzed these passages for instances of outcomes, desired or achieved, in association with home or community gardening. Ultimately, I grouped these outcomes into the four themes identified in the results section. Food Dignity co-investigators in each of the five CBOs have reviewed and approved the findings reported here.

### Team GROW Garden Harvest Measures and Survey

Team GROW (Gardener Researchers of Wyoming) formed a subset of the Food Dignity research with FLV. In 2012, FLV convened five experienced gardeners to ask what garden-related research questions they had. This resulted in the Team GROW endeavor to quantify food production in Laramie home and community garden plots. Between 2012 and 2014, a total of 33 gardening households tending 39 unique plots weighed and recorded each of their garden harvests. Their records included whether they ate, stored, or shared the harvest.

After the pilot year, FLV recruited 31 participants (including three households repeating from 2012) for the 2013 season, actively seeking diversity both in demographics and gardening expertise. In 2014, only gardeners who participated in 2012 and/or 2013 were invited to participate again. Twelve gardeners tending 14 plots measured their harvests again in the 2014 growing season.

In 2015, we also surveyed the gardener-researchers about other outcomes of their gardening. The outcome questions in the survey, listed in Table 1, drew from the garden literature reviewed above and from the input of Team GROW members during annual planning and celebration meetings. We also asked a parallel set of questions about their motivations for gardening. FLV invited all Team GROW gardeners who had participated in any year, whom they could still reach (*n*=28 out of the 33 households), to take the survey. Twenty responded.

Core results from the harvest data are reported elsewhere (Conk & Porter, 2016). In this study, I provide additional outcome detail from that data and analyze results from the 2015 survey.

### Growing Resilience Controlled Trial Feasibility Pilots

By 2012, FLV and BMA had found more community interest in food gardening than they could support with their Food Dignity sub-award funding alone. Building on this interest, the observational literature, and early reports in our case study work about health benefits of gardening, we secured additional funding for a two-site feasibility study to assess health impacts of new home gardens. The research here reports results from these pilots, conducted in 2013. We used a controlled trial design and were guided by a community-university steering committee in each place. We called the pilots *Growing Resilience.*

We recruited 21 households with 29 adult participants total, across the two communities. Nine households with 10 participants were in Laramie, Wyoming, where three people in three households were controls and seven people in six households gardened. In Wind River Indian Reservation, BMA, and tribal health organization, partners recruited 12 households with 19 adults. Eight households were randomized to gardening and four to serve as controls. Thus, in total, one third of the households (14) received garden installation and support from FLV or BMA in 2013. The remaining seven households served as control households (some of whom received garden support the following year). Each CBO recruited these households from their personal and professional networks among those interested in gardening but who did not have a home or community food garden in the past year.

With each adult participant, we sought to measure height and weight, administer a validated quality-of-life survey that assesses mental and physical health (SF-12^®^ Health Survey version 2), and assess hand strength before gardening began (in May 2013) and then at the tail end of the gardening season (in September). The survey included an open-ended opportunity for comment. We were able to gather complete pre- and post-data with all 10 adult participants in Laramie. In Wind River, we collected pre- and post data-for one control adult and six gardening adults; we have only one data point for the remaining 12 participating adults.^1^

We also held focus groups in late 2013, one in Wyoming and one on the reservation, which included representatives from 12 of the 21 participating households. In each group, one person from the University of Wyoming facilitated the group while another took detailed notes that approximated transcription.

For this study, I coded the open-ended survey responses and the focus group notes for outcomes of gardening. In addition, though the sample sizes were much too small to draw any quantitative conclusions, I share some of the pre- and post-results in an anecdotal way.

## Results

By examining the gardening outcome results from the mix of research methods described above, I identified four categories of benefits from food gardening: (1) improving individual health; (2) producing healthy food; (3) providing cultural ecosystem services in recreation, culture and social networks; and (4) fostering healing and transformation.

### 1. Gardens for health

Results from the research projects described here corroborate the growing evidence base that suggests gardening improves health and wellness for gardeners. For example, in the focus groups and post-season surveys conducted as part of the feasibility pilots on health impacts of gardens, new gardening participants in Laramie and on Wind River Indian Reservation reported four types of health benefits:

- *Reduction in medication use for chronic health issues* (e.g., “My blood pressure went down. I’m taking less meds”; “My doctor took me off my anti-depressants… it really made a difference for my depression and my pain levels… taking fewer painkillers.”)
- *Deepened and widened family and social networks* (e.g., “It connected the neighborhood. It became our little mini-community”; “It brought the family closer—everyone wanted to see what was coming from the garden. They’d all be around the kitchen when we were cooking.”)
- *Improved emotional health* (e.g., “It gave me routine and a purpose to be outside in the sunshine. It calmed me”; “It’s just fun. I put my swing right by the garden.”)
- *Improved access to fruits and vegetables* (e.g., “I love fruits and vegetables, but can’t afford it… this is something I can afford”; “It provided more fresh stuff for our family… that really helped our diet.”)

Quantitatively, while the pilot sample was not even close to being powered to detect significant differences, the Laramie pre- and post-data we gathered with all seven gardening and three control adults *might* possibly indicate the gardeners could *possibly* enjoy better outcomes than controls in BMI, hand strength, and mental health. For example, the three control participants gained an average of 4.67lbs. (2.11kg), with a mean BMI increase of 0.57 kg/m^2^. The seven gardeners gained 1.14lbs. (1.52kg) on average, with a 0.2 BMI increase. On the 100-point, 12-item Short Form Health Survey (SF-12) scale for mental health, gardeners improved by seven points on average and controls decreased by one point on average. We have found similar directional (but again, nonsignificant) trends in a second pilot design year with another 10 households in Laramie 2016 (unpublished data), and these results are consistent with the gardens-and-health research reviewed in the introduction. However, our samples were much too small for these numbers to suggest more than the need for further research. We are currently assessing these and other health outcome questions in an RCT with BMA and other partners on Wind River Indian Reservation (Growing Resilience in Wind River Indian Reservation (GR), 2017).

In Team GROW, all 20 gardener-researchers (the Laramie gardeners who had quantified their food harvests) who responded to a survey about the outcomes they experienced from gardening, reported that gardening benefited their health to at least “some extent.” (See Table 1 for full survey results about gardening outcomes.) In addition, their top ranked outcome from gardening was “feeling productive.”

In addition, community-based coinvestigators and participants in the Food Dignity project have described more systemic and community-level yields of gardening that are related to health. I share these in the healing and transformation section below.

### 2. Gardens for high-quality food

Our research indicates that gardeners produce nutritionally relevant quantities of food. In addition, gardeners highly value the quality of the food they produce.

In the Team GROW research, home and community gardeners measured the quantities of food they were growing between 2012 and 2014 in Laramie, Wyoming. Results indicate that the average plot was 253ft^2^ (23.5m^2^) and yielded an average of 128lbs. (58.06kg) f food, or 0.51lbs. (0.23kg) per square foot. The average vegetable harvest was enough to supply two adults with the daily U.S. Department of Agriculture-recommended amount of vegetables for four and a half months (Conk & Porter 2016). This is in spite of Laramie having a challenging high-altitude, windy and semi-arid growing climate (designated as USDA zone 4b, the toughest growing zone in the continental U.S.).

Variation in productivity rates between Team GROW gardeners was enormous. For example, in the 2014 season, harvest rates varied nearly 10-fold between plots (by 967%, ranging from 0.12 to 1.16 lb./ft^2^ [0.59 to 5.66 kg/m^2^]). Within-gardener yield variation, from season to season in the same plot, was much lower, though still substantial, at 39% on average (calculated from the 12 gardeners who participated in more than one season). At the top end, the gardener with the highest yield rate by weight grew 247lbs. (112.04 kg) of food in a 120ft^2^ (11.15m^2^) community garden plot (2.06 lbs./ft^2^ [10.06 kg/m^2^], in 2012). The harvest with the highest economic value, in total and per square foot, was US$2,599 worth of produce (calculated at Laramie Farmers’ Market prices) from a 391ft^2^ home garden (US$6.64/ft^2^, in 2013). This included 145lbs. (65.77kg) of cucumbers valued at US$362 and 255lbs. (115.67kg) of tomatoes valued at US$1,274. Of total harvests recorded by the 31 gardeners participating that season, this particular gardener raised two-thirds of the cucumbers and 35% of the total tomatoes. Also, that year, at the other end of productivity, six gardeners—nearly 20% of the participants that season—had harvest rates under 0.2 lbs./ft^2^ (0.98kg/m^2^).

Quantity aside, producing high *quality* food was a highly valued outcome among gardeners. The Team GROW members who took the survey reported having better quality food and food they know is safe, as two of the four top-ranked outcomes from gardening (see Table 1). In another set of questions about their motivations for gardening in that survey, which mirrored the outcome questions, having better quality food emerged as their top-ranked reason for gardening. Similarly, in interviews and during site visits, gardeners working with the four CBO partners in Food Dignity that support gardens (ENYF, WCP, BMA, and FLV) also mentioned the importance of gardening in yielding quality food. For example, a gardener in eastern New York noted, “all the vegetables, I think, are sweeter,” from her garden than what she can buy in the store. Several people in Wind River discussed how growing their own food helped to avoid “chemicals” in store-bought food. One noted, “the supermarket carrots don’t have hardly any taste but if you taste one that you grow yourself, it’s just like the difference between night and day.” Also, in three of the four communities (with the exception being Ithaca), at least some of the interviewees noted that growing their own food was the best, and sometimes the only, way to get high quality produce.

Even people new to gardening via the Growing Resilience feasibility pilots, who had small gardens (about 80 ft^2^ [7.43m^2^] with BMA and 15–30 ft^2^ [1.39–2.79m^2^] with FLV, in accordance with steering committee advice and gardener preferences) and struggled with multiple growing challenges, felt that their gardens gave them meaningful amounts of food. For example, in addition to the comments cited above about improved access to fruits and vegetables, participants reported that “it gave me fresh vegetables for my family that I grew and saved me money” and “I can reduce my food cost.” As one ENYF gardener who was looking forward to retiring put it, “the main reason for it is the quality of the food and if you’re retired, you’re not going to have the income, so financially it’s going to help. You’re not going to have to buy all those foods.”

### 3. Gardens for “cultural ecosystem services”

The sections above report health and food provisioning ecosystem services provided by gardens. This section focuses on “cultural ecosystem services” that gardens may provide through recreation, continuation and expansion of cultural and spiritual traditions, and development or deepening community networks.

#### Growing recreation and aesthetic enjoyment

Gardeners connected with these action research projects talk about gardening, at least in part, as recreation. Growing Resilience gardeners described the pleasure their gardens gave them, saying, for example, “walking down those steps, digging in the dirt, having a great time watering, watching the bees, I’m just in love with those silly bees. I kept my yard cleaner too.” Another noted that gardening is “something you have to do, but you don’t feel like you have to.” In the survey of Team GROW gardeners, 19 out of 20 said they experienced “leisure or pleasure” from gardening to at least some extent (Table 1).

In addition, the community gardens and other public food growing spaces supported by the CBOs draw not only gardeners, but also garden and farm visitors who watch the produce develop over the season, enjoy the flowers, and/or learn about the foods people grow. For example, a visitor to FLV said she had walked by their building regularly just to monitor the progress of pumpkins being grown to share with the Laramie community, and appeared to be a little disappointed when they were harvested. FLV, DDF and ENYF in particular, regularly receive formal requests for tours and, collectively, host hundreds of visitors each year who want to admire and learn from their work.

#### Growing culture and spirit

BMA on Wind River Indian Reservation is helping community members restore traditional varieties of Indian corn and reestablish chokecherries. Gardeners supported by ENYF in Brooklyn grow culturally important foods such as callaloo, long beans, bitter gourd, and hot bonnet peppers. Gardeners in both places help anchor local farmers markets, providing not only fresh produce in general, but diverse varieties that would not otherwise be available for purchase. One gardener who sells at the ENYF market noted that “things that sell like hot bread in the market is callaloo. You cannot plant enough callaloo.” These outcomes include, not only maintaining cultural food traditions, but also sharing them. For example, through their Food Dignity connections, a Jamaican gardener in East New York grew Indian corn from Wind River seeds. Some gardeners in the feasibility pilot studies about gardening appreciated learning about vegetables that were new to them, one saying, “who would have thought I would fall in love with bok choy?”

#### Growing people and relationships

Gardeners report sharing and exchange harvests, labor, and knowledge with their communities. This sharing is likely one of the core means by which gardening deepens social networks and connections.

In Team GROW, the gardener-researchers, who tracked whether they ate, stored, or shared each harvest, shared 30% of what they grew with others (Conk & Porter, 2016). Those who responded to the survey also reported “sharing food with others” as both a motivation for and an outcome of gardening (Table 1).

In interviews, many gardeners talked about sharing food, exchanging knowledge, and offering and receiving physical assistance with gardening labor. Several described, not just what they gave, but also what they receive by sharing. For example, one experienced gardener said that inspiring and mentoring people to grow their own food, “just makes me feel so good.” She also noted the physical help she gets when people come to visit her garden, noting, “I wish that I’d had more people come out. One thing that helps me, is I can’t do all the physical stuff very well anymore, but it passes [knowledge] on and I like to pass on my passion.” Another person reported that someone who shared her land for growing food for the community felt, “glad that she could provide something. She doesn’t have a lot of resources but she has this yard so she was glad that she could use that yard to benefit others and to have that be a resource.” Some gardeners in the feasibility pilots reported with pride, being consulted about gardening; for example, “I had people asking me, how do you do this? What did you use? I’m the expert on raised gardens now, of my friends.”

Some gardeners in the feasibility studies who struggled with depression, physical movement limitations, or both, reported that their new gardens gave them a reason to get up in the morning, noting, “it got me on a better sleep schedule” and “it got me out of bed.” When one person in the Laramie, Wyoming focus group said that, “I spent more time outside than I ever have,” another replied, “wasn’t that neat?” and a third confirmed “me too!” They talked about children coming over to point out new growth or study bugs in the gardens, and friends and neighbors coming over to eat from their gardens or even just to admire them; for example, “my friends came over, and sat on the patio and looked at the garden while we ate. People just really liked it. It was pleasant. We had lunch, we picked fresh basil, made sandwiches.”

Also, several gardeners most involved with the Ithaca, Wind River Reservation and Laramie-based CBOs (WCP, BMA and FLV, respectively) have described the local collaboration teams in these action-research projects as feeling like family. One of the Team GROW gardener-researchers said that the project had connected her with “my people.” All but one of the Team GROW survey respondents noted that meeting other community members was at least a partial outcome from their gardening.

An organizer on Wind River Indian Reservation describes how gardening also helps to educate children (Potter, 2015), which illustrates results from the small subset of Team GROW survey respondents (4 out of 20) who had children at home, who unanimously ranked teaching their children as an outcome from gardening (Table 1). Both WCP and ENYF intentionally build inter-generational relationships by matching teens with local elders who provide mentorship while receiving help with their gardening (Brangman, 2017; Daftary-Steel & Gervais, 2015).

These “cultural ecosystem services” create foundations for and contribute to the last category of outcomes from gardening found in this study: individual and community healing, and transformation.

### 4. Gardens for healing and transformation

The five CBOs collaborating in these food system action research projects both report and aspire to individual and collective healing and transformation with their communities. They intentionally design their community food growing and growing support activities to help reach these goals (Porter, 2018a, this issue), as articulated in their collaborative pathway models (Hargraves & Denning, 2017). They also particularly aim to support people and communities who suffer the most and offer expertise derived from lived experience with food injustice and food insecurity.

BMA and FLV partnered in the feasibility pilots as part of intentionally using home gardens as a strategy for helping people on Wind River Indian Reservation and in Laramie, Wyoming, increase control of their lives and their physical health. A gardener supported by FLV said, “I never would have attempted a garden without this. It wasn’t a possibility. Without this, it would have never happened.” Another also said, “I never would have had a garden. I wouldn’t have gardened at all without this project.” A third mentioned she could not get down on her knees to tend her garden, so it was the raised boxes that FLV provided that made it possible for her to grow food. More broadly, at the start of the Growing Resilience pilots, the head of a tribal health organization collaborating with BMA and me said he approved of the gardening project idea, because, “we need to put health back into the hands of the people.” Similarly, an expert gardener working with ENYF noted that she and other gardeners feel that, “growing, sharing and selling fresh food, growing stuff and selling it to the community, it’s making the community healthier. It’s making us, me, mentally healthier, because people see that this comes from the heart, it’s going here.”

Achievement of such transformative outcomes is challenging to assess or attribute, but the results from these action-research projects do illustrate some examples of how gardening and other forms of community food production have contributed to fostering health and transformation.

Several of the digital stories composed by some Food Dignity partners to share their individual journeys in food justice work vividly illustrate these themes of growing food for healing and transformation. For example, two men who worked as farmers at DDF entitled their stories, “Fresh Start,” and, “My New Life,” with each describing how growing food offered pathways away from jail or prison (Rucker, 2015; Silva, 2015). The availability of these paths was no accident; their boss, a captain in the Alameda County Sherriff’s Department who co-founded DDF, entitled his story, “When Good Food Makes for Good Policing” (Neideffer, 2015).

Some gardeners have planted to regain control of their health and to heal. One gardener began growing her own food to recover her health after becoming highly chemical sensitive from exposure to pesticides (Dunning & Owens, 2016). Another says she planted gardens to take root, more figuratively, in a new community (Dunning, 2015). One participant in the feasibility pilot about health impacts of gardens reported that gardening saved her life. Several gardeners on Wind River Indian Reservation talked about growing their own food to take control over their diabetes and to prevent their children from being diagnosed by building healthy lifestyles, in addition to providing well for their families overall.

For some, gardening also appeared as a gateway to improving their communities and increasing personal influence. One young DDF farmer, in conversation with me, marveled at the power he had to physically change his community after being part of transforming a corner lot from an eyesore into a beautiful and productive garden. A person who became a gardener with help from FLV via the feasibility pilot, later went to his first city council meeting to support providing public land for a proposed FLV community farm. While there, earlier in the agenda, he spoke powerfully in favor of locating a recreation facility on the west side of Laramie, which is literally and figuratively on the other side of the tracks from the city center. Similarly, that was also my first Laramie city council meeting, and though there to support the farm proposal, I also spoke up on an earlier agenda item, in favor of aquifer protection. In this way, our involvement with FLV also led us to become more active citizens, and to speak up in this formal policy-making setting. Leaders at ENYF talk about people in their communities dedicated to growing food to take back empty lots, beautify their worlds, and feed their neighbors (Daftary-Steel, 2015; Marshall, 2015; Vigil, 2015). Others describe how growing food contains transformational lessons about having, “the grace to receive” (Dunning 2015) and heeding calls for environmental healing (Brangman, 2015). Other stories are about viewing, acting, and being in our world in a transformed way (Daftary-Steel, 2015), including, as another storyteller concludes, “once you start to see the potential in the people and the place, you can’t help but look for that everywhere you go” (Vigil 2015).

## Discussion

Results from this research confirm and expand upon previous work showing that benefits of food gardening include: improving individual health; producing nutritionally meaningful amounts of quality food; providing cultural ecosystem services in recreation, culture and social networks; and fostering healing and transformation. This array of positive outcomes suggests that supporting home and community food gardening offers an effective public health and sustainable community development strategy.

Understanding more about why and how gardening produces these outcomes, and for and with whom, would inform how to best deepen and broaden these and other positive impacts. To begin outlining future action and research agendas in this arena, I draw from the results presented in this paper and from previous research to discuss potential mechanisms.

### Health, Food, and Gardening

For some of the individual health benefits associated with gardening, mechanisms that likely produce them seem obvious. For example, being physically active and reducing sedentary time are known to improve overall wellbeing (Kohl et al., 2012; Warburton, Nicol, & Bredin, 2006) and gardening inherently entails activity. The link between producing vegetables and increased access to and consumption of them seems transparent. Spending time outside is known to improve mental health and gardening requires being outdoors (Ryan, Weinstein, Bernstein, Brown, Mistretta, & Gagné, 2010). In addition, several gardeners here reported that their gardens draw them to sit outside even when not actively gardening. Why being outside improves emotional health is less certain, though one plausible mechanism is that sun exposure improves vitamin D levels, while inadequate levels are associated with depression (Penckofer, Kouba, Byrn, & Estwing Ferrans, 2010). An additional theory involves exposure to mood-improving microbes that are common in soil (Reber et al., 2016), which may more easily transfer to humans via gardened foods than via store-bought foods (Bryce, 2013).

The power conveyed by becoming a producer, as opposed to only a consumer, may also improve well-being; self-determination theory suggests that feelings of autonomy and control contribute to health (Deci & Ryan, 2008). Also, gardeners report feeling productive, which is associated with a higher quality of life (Kim, 2013; Litt, Schmiege, Hale, Buchenau, & Sancar, 2015), especially when the productive activity also benefits others (Aknin et al., 2013; Matz-Costa, Besen, Boone James, & Pitt-Catsouphes, 2014). Some of the gardeners in this study have said that sharing their food and their knowledge has enhanced their own well-being. This is in addition to the benefits of increased family and community feelings of connectedness found in this and previous research.

Though some of the health benefits observed in association with gardening maybe be only that—correlated but not causal, simply indicating that healthier people are more likely to garden—the feasibility pilots reported here and in the 22 studies in the meta-analysis review (Soga, Gaston, & Yamaura, 2017) all involved pre- and post-health outcome measures. This time order, of hypothesized cause before effect, adds to the plausibility of gardening positively affecting health. Also, arguably, if a person reports that gardening makes them feel healthier, as so many in this and other studies do, then their subjective well-being is indeed improved by definition. If a survey used to measure well-being (such as the SF-12 used in these feasibility pilots) does not capture this improvement, then this is a failure of the instrument.

### Healing, Transformation and Gardening Support

The array of potential causal pathways for health and food benefits of gardening discussed above, if real, would suggest that such benefits would accrue to gardeners at large, even those who do not receive technical assistance or associate with food justice CBOs that support such food production. This would also likely be true for many of the recreational services that gardening provides. However, it seems plausible that the, “growing people and relationships,” outcomes, and moreover, “healing and transformation,” ones, would be enhanced by the support strategies the five CBOs use. Moreover, CBOs extend these benefits to people who wish to garden but could or would not without such support. Because all of the gardeners in this research were associated with the work of food justice CBOs, I can only hypothesize from our observations about how these associations may have impacted distribution and depth of these gardening outcomes.

The broad set of benefits in culture and spirit, people and relationships, and healing and transformation reported here, appear to be entwined with and emerging from the CBOs’ strategies for supporting gardening and gardeners. As described elsewhere (Porter, 2018a, this issue), these CBOs extensively use organizing strategies to achieve transformational goals with their communities. Technical support for gardening, such as that traditionally provided by cooperative extension agencies in the U.S. and also included in activities of these CBOs, simply aims to help improve gardeners’ skill levels for greater food production. However, rather than as an end in itself, the CBOs view gardening as a strategic activity that provides one of many means to larger ends of community health, food security, equity, and power. These CBOs intentionally enable gardeners to also become vendors, farmers, mentors, donors, policy advocates, educators, grantees, grantors, and more, if and as they wish to. They also help enable people to become gardeners, or even farmers, if they wish to. As two food justice activist scholars note, “no amount of fresh produce will fix urban America’s food and health gap unless it is accompanied by changes in the structures of ownership and immigration laws and a reversal of the diminished political and economic power of the poor and lower working-class” (Holt Giménez & Shattuck, 2011, p. 133). As articulated in their collaborative pathway models (Hargraves & Denning, 2017), all five CBOs aim to increase political and economic power of people who currently have the least, including via supporting community-based food production such as gardening. As the authors of case studies with four community gardens in eastern London argue, such gardens create, “contexts for effective community mobilization… opening up new possibilities for being, seeing and doing” (Guerlain & Campbell, 2016, p. 220). The intentionality in creating these spaces leads Pudup (2008) to argue that community gardens should instead be called organized garden spaces (see also Saldivar-Tanaka & Krasny, 2004).

As a public health nutrition scholar, when I present results from this work about health benefits of gardening, I have reason to fear that I am framing gardening as another health-behavior-change imperative: not only should people eat more fruits and vegetables, they should grow them. A scientist in the audience at one seminar, who also identified as a single mother, asked wearily, “when do I get to rest?” However, the CBO organizers appear to be agnostic about whether community members become gardeners at all; they focus on people and community, not on production or even food more generally (Porter, 2018a, this issue). For example, after the Growing Resilience feasibility pilots, FLV engaged with me to redesign our approach to enable people to set their own health improvement goals and then choose how to reach them, rather than randomly assigning people to gardening. By offering multiple ways for community members to engage, these CBOs model what Guerlain and Campbell describe as better accounting “for what participants themselves would like to achieve in their own lives, rather than in relation to externally imposed notions of what counts as political change” (2016, p. 220).

That said, when people do wish to garden, four of the five CBOs (one focuses on community farming and does not engage directly in gardening activities) strive to support and enable them to do so (Porter, 2018a, this issue). The full gardening support and installation “packages” that FLV and BMA provide have almost certainly enabled more people to garden. As reported above, a few of the FLV gardeners have said explicitly that they would never have been able to garden without that help. Also, the community gardening spaces that ENYF, WCP, and FLV have cultivated offer the space, soil and, especially with FLV in Laramie, affordable water, that are all necessary for gardening but not everyone has access to. Results from another study within the Food Dignity project, where US$40 gardening mini-grants were randomly provided to half the attendees at a gardening workshop, found that even small amounts of material support spurred interested people to start or expand food gardens (Porter, McCrackin, & Naschold, 2016).

### Future Research

Results from the three randomized controlled trials currently underway will substantially improve the quality, quantity, and specificity of evidence for how gardening impacts individual health outcomes. If these studies find positive results, the next question would be about if and how much the quantity of food produced—in total and as rate per area—is related to health outcomes. Based on our qualitative observations and gardener insights, I would hypothesize that most of the physical and mental benefits are not closely tied to productivity, as long as a harvest does not fail entirely.

In links between healing, transformation, and gardening support, it seems plausible that technical assistance alone would likely help gardeners to improve yield quality and quantity. The enormous range of harvest rates found in Team GROW certainly indicates that there is room for such increases. In addition, technical support would help urban gardeners avoid and mitigate heavy metal exposure risks that gardening in contaminated soil poses (Al-Delaimy & Webb, 2017). However, such narrow and limited forms of support are unlikely to enable people, particularly those who face physical, financial, and/or land access challenges, to begin growing their own food in the first place. Technical assistance alone also would not, plausibly, work to connect gardeners more directly and deeply with one another and with other food system activities (e.g., sharing, selling, advocating, mentoring) the way the CBOs’ strategic activities aim to (Porter, 2018a, this issue). The social healing and transformation outcomes, and potential outcomes, of gardening may hinge upon the kinds of community organizing strategies that the food justice CBOs use (Porter, 2018a, this issue).

## Conclusion

The gardening outcome data from the Food Dignity case stories, Team GROW project, and Growing Resilience feasibility pilots, confirm and expand findings from previous research which indicate that gardening improves health, produces nutritionally meaningful quantities of quality food, and provides important cultural ecosystem services (such as recreation, cultural enrichment, and community building). In addition, perhaps especially because of the strategies employed by food justice CBOs that collaborated in this research, gardening activities have also yielded individual and social healing and transformation.

Arenas ripe for future research on impacts of gardening include further quantifying and specifying individual health changes and causality, assessing relationships between garden productivity and outcomes, and further documenting and evaluating community-level outcomes. Another action research priority is trialing and assessing strategies for maximizing access to gardening and for maximizing positive outcomes from gardening via policy, technical, and community-organizing forms of support. In the meantime, however, the growing evidence for multiple benefits of home and community gardening suggests the wisdom of enabling anyone who wishes to start growing some of her own food to plant some seeds.

## Figures and Tables

**Table 1:** Team GROW Survey Responses to the Question “To what extent does your food gardening actually result in these outcomes (regardless of whether or not they are motivating factors for you)?” Results below denote the percent of respondents and (number of respondents) for each “extent” rank. Items are listed in decreasing order of respondent ranking (by the sum of “to a moderate extent” or higher answers).

		Not at all	To some extent	To a moderate extent	To a great extent	To a very great extent	*Respondent total #*
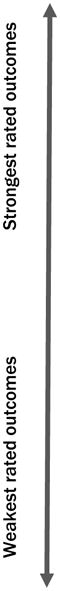	I taught my kids about gardening (leave blank if you do not have children at home)	0%	0%	0%	50%	50%	*4*
	I felt productive	0%	0%	30%	40%	30%	*20*
	I had better quality food	0%	5%	5%	50%	40%	*20*
	I grew food that I knew was safe	0%	5%	25%	20%	50%	*20*
	I shared food with others	5%	5%	35%	30%	25%	*20*
	I experienced leisure or pleasure	5%	5%	15%	30%	45%	*20*
	I was more self-sufficient	0%	16%	26%	16%	42%	*19*
	I spent time outdoors	0%	10%	20%	35%	35%	*20*
	I reduced my stress	10%	10%	25%	20%	35%	*20*
	I increased my physical activity	0%	25%	25%	20%	30%	*20*
	I improved my health	0%	25%	25%	30%	20%	*20*
	I saved money on food	5%	25%	40%	20%	10%	*20*
	I met other community members	5%	35%	15%	25%	20%	*20*
	I ensured my household had enough to eat	15%	30%	25%	15%	15%	*20*

## Footnotes

1 Based on this experience, we completely redesigned our data-gathering approach in the full-scale Growing Resilience randomized controlled trial currently underway in Wind River Indian Reservation (University of Wyoming et al., 2016). Instead of scheduling data-gathering appointments with participants at their homes, households came to a central data-gathering location, with transportation provided as needed and stipends provided. The project also now includes more substantial partnerships with the tribal health organizations involved than during the feasibility pilot. So far, in our first two years of the full-scale study, we have had excellent participant retention and return rates.
